# Pregnancy in women with liver cirrhosis is associated with increased risk for complications: A systematic review and meta‐analysis of the literature

**DOI:** 10.1111/1471-0528.17156

**Published:** 2022-03-31

**Authors:** Laurine L. van der Slink, Irma Scholten, Faridi S. van Etten‐Jamaludin, Robert B. Takkenberg, Rebecca C. Painter

**Affiliations:** ^1^ Department of Obstetrics and Gynaecology, Amsterdam Reproduction and Development Amsterdam UMC, University of Amsterdam Amsterdam the Netherlands; ^2^ Department of Obstetrics and Gynaecology Deventer Ziekenhuis Deventer the Netherlands; ^3^ Medical Library Academic Medical Centre Amsterdam UMC, University of Amsterdam, Research Support Amsterdam the Netherlands; ^4^ Department of Gastroenterology and Hepatology, Amsterdam Gastroenterology Endocrinology Metabolism Amsterdam UMC, University of Amsterdam Amsterdam the Netherlands

**Keywords:** caesarean section, liver cirrhosis, meta‐analysis, mortality, pre‐eclampsia, pregnancy, preterm delivery, variceal haemorrhage

## Abstract

**Background:**

Pregnancy and liver cirrhosis is a rare but increasing combination. Liver cirrhosis can raise the chance of maternal and fetal mortality and morbidity, although the exact risks remain unclear.

**Objective:**

To provide a systematic literature review and meta‐analysis on maternal, fetal and obstetric complications among pregnant women with liver cirrhosis.

**Search strategy:**

We performed a systematic literature search in the databases PubMed/MEDLINE and EMBASE (Ovid) from inception through 25 January 2021.

**Selection criteria:**

Studies including pregnancies with liver cirrhosis and controls were eligible.

**Data collection and analysis:**

Two reviewers independently evaluated study eligibility. We used the random effects model for meta‐analysis.

**Main results:**

Our search yielded 3118 unique papers. We included 11 studies, including 2912 pregnancies in women with cirrhosis from 1982–2020. Seven studies were eligible for inclusion in the meta‐analysis. The overall maternal mortality rate was 0.89%. Maternal mortality and variceal haemorrhage were lower in recent than in older studies. Most cases of maternal mortality due to variceal haemorrhage (70%) occurred during vaginal delivery. Pregnant women with liver cirrhosis had a higher chance of preterm delivery (OR 6.7, 95% CI 5.1–9.1), caesarean section (OR 2.6, 95% CI 1.7–3.9), pre‐eclampsia (OR 3.8, 95% CI 2.2–6.5) and small‐for‐gestational‐age neonates (OR 2.6, 95% CI 1.6–4.2) compared with the general obstetric population. Subgroup analyses could not be conducted.

**Conclusions:**

Liver cirrhosis in pregnant women is associated with increases in maternal mortality and obstetric and fetal complications. Large international prospective studies are needed to identify risk factors for unfavourable outcome.

**Tweetable abstract:**

Systematic review and meta‐analysis: higher risks that pregnant women with liver cirrhosis face are quantified.

## Tweetable abstract

Systematic review and meta‐analysis: higher risks that pregnant women with liver cirrhosis face are quantified.

**Linked article**: This article is commented on by Orene Greer, pp. 1653 in this issue. To view this minicommentary visit https://doi.org/10.1111/1471-0528.17155.

## 1 INTRODUCTION

Pregnancy is a rare event in women with liver cirrhosis in part due to decreased natural fertility rates.^1^, ^2^ The prevalence of cirrhosis in women of reproductive age is 0.045%.^3^ The incidence of cirrhosis in pregnancy is reported as approximately 1 per 4500 pregnancies.^4^, ^5^ Worldwide, the most common causes of liver cirrhosis in women are viral hepatitis, autoimmune hepatitis, alcoholic liver disease and non‐alcohol‐related fatty liver disease.^1^ Although pregnancy and cirrhosis is a rare combination, it appears to have become more frequent in the last decades possibly owing to improvements in the treatment of liver cirrhosis, increased awareness of cirrhosis after the introduction of screening in high‐risk populations, and the availability of assisted reproductive techniques.^6^, ^7^, ^8^ For clinicians and patients alike, when deciding on family planning and obstetric management, the effects of liver cirrhosis on the course of pregnancy and the effects of pregnancy on underlying cirrhosis are both of importance.

Previous studies have shown that pregnancy in women with cirrhosis is associated with a high risk of complications, including maternal and perinatal mortality.^3^, ^9^ However, studies are small and report on a variety of outcomes.^5^, ^10^, ^11^, ^12^, ^13^, ^14^ This makes it difficult to advise both patients with liver cirrhosis and their clinicians on the course of a planned pregnancy. A systematic review and meta‐analysis of the literature could provide with robust estimates of the risks involved in pregnancy. Here, we provide a systematic review reporting on the maternal, fetal and obstetric complications among pregnant women with liver cirrhosis.

## 2 METHODS

The protocol of this review was registered in PROSPERO (CRD42018080575) on 1 December 2017. We followed the Preferred Reporting Items for Systematic Reviews and Meta‐Analysis (PRISMA) statement and the Meta‐analysis of Observational Studies in Epidemiology (MOOSE) group. None of the authors received specific funding for this review. There was no patient or public involvement in the development or implementation of this review.

### 2.1 Search strategy

A literature search was performed in the databases PubMed/MEDLINE and EMBASE (Ovid) from inception to 25 January 2021. The search had no language restrictions. We used medical subject headings as well as title and abstract terms and word variants of pregnancy and liver cirrhosis. Appendix S1 presents the complete search strategy.

### 2.2 Study selection process

We selected cohort studies and case control studies with patients who had liver cirrhosis and were pregnant. Diagnoses of liver cirrhosis were considered confirmed when based on biopsy findings or on imaging combined with laboratory findings. Cohorts or case control studies had to include a minimum series of five patients with liver cirrhosis. Results had to contain at least one item of one of the designated outcome domains (see heading ‘Outcome measurements’). We included studies in the meta‐analysis if they contained a control group. When our search found papers which analysed identical patient populations multiple times and which reported on identical outcomes, we selected the most recent or most relevant study, and excluded the other studies reporting on the same population. Other exclusion criteria were non‐human studies, studies outside pregnancy and case reports, case series, opinions or reviews.

Two reviewers (LLS and RBT) independently screened the titles and abstracts of studies retrieved by the database searches. We requested the full text if a study was potentially relevant and performed independent screening to assess eligibility *in duplo* on full text. Disagreement about including a study were resolved through discussion or by consulting a third reviewer (RCP).

### 2.3 Quality assessment

We used the Newcastle–Ottawa scale for cohort studies for quality assessment and estimating the risk of bias. Two reviewers (LLS and IS) independently assessed the included studies. We did not exclude studies based on poor quality.

### 2.4 Outcome measurements

The outcomes of interest were divided into three domains: maternal complications, obstetric complications and fetal outcome or complications. Maternal complications included maternal mortality, hepatic decompensation, gastrointestinal bleeding or variceal bleeding. We defined maternal mortality as maternal death during pregnancy or within 42 days of termination of pregnancy.^15^ We defined hepatic decompensation as the presence of jaundice, ascites or hepatic encephalopathy.

Obstetric complications included caesarean delivery, preterm delivery (birth <37 completed weeks’ gestation), postpartum haemorrhage (loss of >500 ml blood within 24 h during or after delivery), miscarriage (pregnancy loss before 20th week), placental abruption and hypertensive disorders of pregnancy (including pregnancy induced hypertension (PIH; systolic blood pressure ≥90 mmHg or diastolic blood pressure ≥140 mmHg), pre‐eclampsia (hypertension with proteinuria >300 mg per 24 hours) and the syndrome of haemolysis, elevated liver enzymes and low platelet count (HELLP; diagnosed by laboratory abnormalities)).

Fetal complications included neonatal mortality (death within 28 days after pregnancy), admission to neonatal intensive care unit, intrauterine fetal demise (pregnancy loss at or after 20th week of gestation), congenital malformations and small‐for‐gestational age (SGA, birthweight less than 10th percentile for gestational age). Low birthweight (birthweight <2500 g) was used when SGA was not reported.

### 2.5 Data extraction

Two authors (LLS and IS) independently extracted data from the included studies using a predefined and structured data extraction form created for this systematic review. In addition to the outcomes of interest, we extracted the study characteristics (authors, year and journal of publication), study design, inclusion and exclusion criteria, number of patients and number of pregnancies with cirrhosis and, if appropriate, number of controls of all included studies. In studies missing data, we contacted first authors at least two times to request these data.

### 2.6 Data analysis

We performed a meta‐analysis, using a random effects model, if at least three studies reported on the outcome of interest. Odds ratios (OR) and 95% confidence intervals (CI) comparing women with liver cirrhosis with controls were calculated. A *P*‐value <0.05 was considered statistically significant. We used *I* ^2^ for testing statistical heterogeneity.^16^ Sensitivity analyses were performed when the *I* ^2^ test showed evidence of high heterogeneity, corresponding to *I* ^2^ >75%. We performed sensitivity analyses by removing contributing papers from the analysis that were thought to be responsible for heterogeneity based on deviating study design, case definition, study population and aetiology of liver cirrhosis. We used The Cochrane Manager Reviewer (REVMAN 5)^17^ for the statistical analysis.

To analyse decreases in maternal mortality and variceal haemorrhage, we calculated the odds ratio of the total events that occurred, after dichotomising the odds ratios from studies conducted before and after the total mean study period of all included studies. We also made time sequenced plots to visualise the decreases of maternal mortality and variceal haemorrhage. We used the median date of inclusion of individual studies as the value on the *X*‐axis and the incidence rates as value on the *Y*‐axis.

## 3 RESULTS

**Figure 1:**
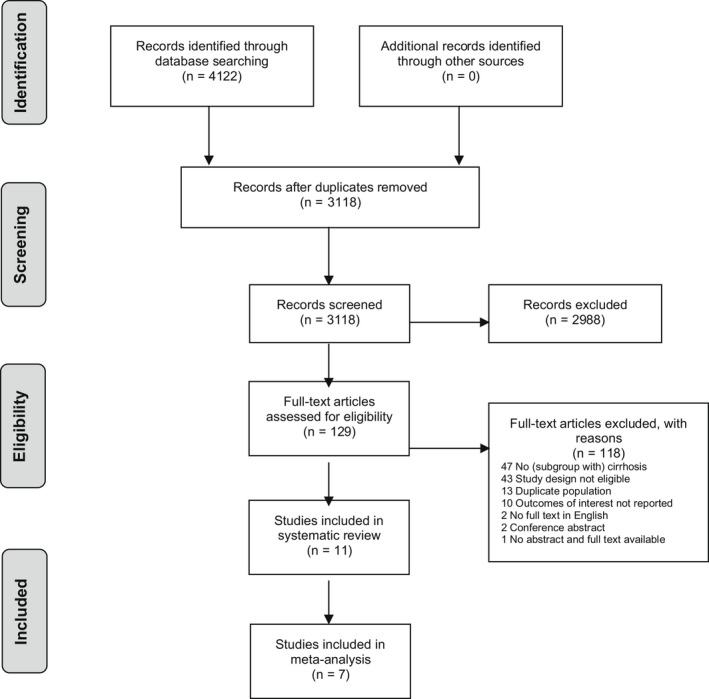
Flow chart of study selection

Our search retrieved 3118 unique publications. After title and abstract screening, 130 studies were selected for full text eligibility screening. We included 11 studies (with 2901 pregnancies with liver cirrhosis) in the systematic review, of which seven were eligible for the meta‐analysis (Figure 1). The total number of pregnancies with liver cirrhosis included in the meta‐analysis was 2685, as well as 4 283 173 pregnancies without liver cirrhosis (control group). Table 1 describes the general characteristics of included studies. All retrieved studies were cohort studies. The aetiologies of liver cirrhosis in the included studies corresponded with the most common aetiologies of liver cirrhosis in women worldwide.

**Table 1:** Characteristics of included studies

First author	Year of publication	Place	Study design and origin of data	Study period	Quality assessment	Number of pregnancies with cirrhosis	Number of pregnancies without cirrhosis	Included in meta‐analysis
Salman^18^	2020	Cairo, Egypt	Prospective cohort; Cairo University hospitals	2013–2018	Fair	100	100	Yes
Flemming^19^	2020	Kingston, Canada	Retrospective cohort; Multiple databases housed in the Institute for Clinical Evaluative Sciences	2000–2017	Good	2098	10 110	Yes
Hagström^20^	2018	Stockholm, Sweden	Prospective cohort; Swedish Medical Birth Register	1997–2011	Good	103	1 361 566	Yes
Jena^21^	2017	Bangalore, India	Retrospective cohort; St. John Medical College	2010–2015	Fair	28		No
Palatnik^4^	2017	Milwaukee, USA	Retrospective cohort; Medical College of Wisconsin	2005–2016	Good	31	124	Yes
Danielsson Borssén^22^	2016	Umeå, Sweden	Retrospective cohort; questionnaire	1999–2010	Poor	43		No
Puljic^23^	2016	San Diego, USA	Retrospective cohort; databases of California Department of Health Services	2005–2009	Good	37	2 248 218	Yes
Rasheed^24^	2013	Sohag, Egypt	Prospective cohort; Sohag University Hospital	2009–2012	Good	129	647	Yes
Westbrook^25^	2011	London, UK	Prospective cohort; King's College Hospital	1984–2009	Fair	62		No
Murthy^26^	2009	Toronto, Canada	Retrospective cohort; Nationwide Impatient Sample database	1998–2005	Fair	187	662 408	Yes
Britton^27^	1982	Portland, USA	Retrospective cohort; Maine Medical Center	Unknown	Fair	83		No

### 3.1 Quality assessment

Six studies were assessed as good quality, four studies as fair quality and one study as poor quality (Table S1). With one exception,^22^ all studies scored at least two out of four stars for selection criteria and two out of three stars for outcome criteria. The most frequent source of risk of bias included ‘no control group’ (four studies did not include a control group), ‘selection of controls’ and ‘adequacy of follow‐up’, as nine studies were retrospective cohorts.

### 3.2 Maternal complications

We present only descriptive data on maternal complications, as we could not conduct a meta‐analysis for maternal complications due to a lack of odds ratios in the published reports on this topic. Maternal death rate was reported by eight studies and ranged from 0% to 7.8%. In total, 25 events of maternal deaths in 2794 pregnancies of women with cirrhosis (0.89%) were recorded, compared with 0.010% in control pregnancies (included studies mentioned 197 events in 2 024 845 controls), which is equivalent to an 80‐fold higher rate (OR 80.2, 95% CI 27.3–235.1; *P* < 0.0001). The total rate of maternal deaths in women with liver cirrhosis in older studies (year range 1982–2002) was higher than the rate in recent studies (year range 2004–2016) (OR 2.9, 95% CI 1.2–7.1; *P* = 0.02). This trend in decreasing maternal mortality over time is illustrated by the time sequenced plots of the incidences of included studies (Figure S1). The most common cause of maternal death was variceal haemorrhage (*n* = 13), the majority of which occurred during vaginal delivery (*n* = 9), and some during pregnancy (*n* = 2), during caesarean section (*n* = 1) or in the postpartum period (*n* = 1). Other causes of maternal mortality were sepsis (*n* = 2), pre‐eclampsia (*n* = 1), hepatic decompensation (*n* = 2), flare of autoimmune hepatitis (*n* = 1) and unknown (*n* = 6) (Table S2).

Variceal haemorrhage, either resulting in maternal death or not, occurred 113 times in 2858 pregnancies (4.0%) reported in 10 studies. In most cases the occurrence of variceal haemorrhage during pregnancy was new and had not occurred preconceptionally. The rate of preconceptional oesophageal varices was mentioned in none of the included studies. In total, 12% of variceal haemorrhage ended in maternal death (*n* = 13). The total rate of variceal haemorrhage in women with liver cirrhosis in older studies (year range 1982–2002) was higher than the rate in recent studies (year range 2004–2016) (OR 2.7, 95% CI 1.7–4.1; *P* < 0.0001), as is illustrated in the time sequenced plot of incidences of variceal haemorrhage (Figure S2). In the study of Rasheed et al.,^24^ 47% of pregnancies were complicated by variceal haemorrhage. The remaining nine studies found lower percentages of variceal haemorrhage varying between 0% and 16%.^4^, ^18^, ^19^, ^20^, ^21^, ^23^, ^25^, ^26^, ^27^ Screening endoscopy for oesophageal varices during pregnancy was reported in five recent studies with rates of 3%,^19^ 17%,^20^ 28%,^18^ 35%^4^ and 60%.^25^ Accompanying endoscopic therapy prior to or during pregnancy for (non‐bleeding) oesophageal varices was reported in three studies with rates of 10%,^20^ 18% ^21^ and 23%.^4^

The incidence of decompensated liver cirrhosis varied between 2% and 21%,^19^, ^21^, ^23^, ^25^ with an outlying incidence in the study of Rasheed et al.,^24^ who reported 64%. Ascites prevalence was reported in five studies and ranged from 3% to 11%.^4^, ^18^, ^24^, ^25^, ^26^ Hepatic encephalopathy was also reported in five studies and ranged from 1% to 13%.^4^, ^18^, ^24^, ^25^, ^26^

### 3.3 Meta‐analysis on obstetric and fetal outcomes

**Figure 2:**
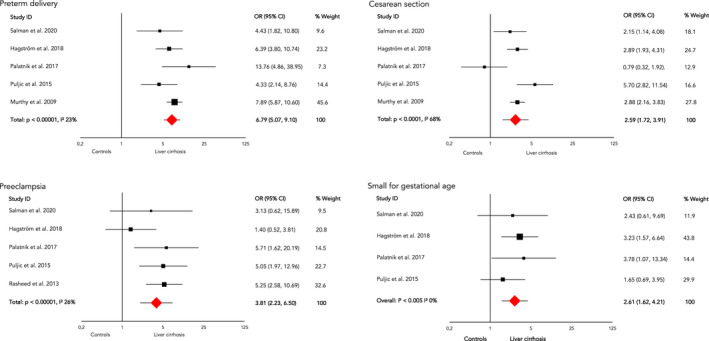
Meta‐analysis

Meta‐analysis showed a significantly higher chance on preterm delivery (OR 6.7, 95% CI 5.1–9.1), caesarean section (OR 2.6, 95% CI 1.7–3.9), pre‐eclampsia (OR 3.8, 95% CI 2.2–6.5) and SGA neonates (OR 2.6, 95% CI 1.6–4.2) in pregnant women with liver cirrhosis compared with pregnant women without liver cirrhosis (Figure 2). There was high heterogeneity in the analyses of preterm delivery, caesarean section and SGA. The studies thought to be responsible for most heterogeneity were those of Rasheed et al.^24^ and Flemming et al.,^19^ based on their study design, patient characteristics and inclusion criteria (see Discussion). Sensitivity analyses after elimination of these two studies did lower the *I* ^2^ on the outcomes preterm delivery (*I* ^2^ before 94%, after 23%) and SGA (*I* ^2^ before 71%, after 0%). The sensitivity analysis of caesarean section maintained moderate heterogeneity (*I* ^2^ before 95%, after 68%).

### 3.4 Obstetric and fetal complications

Table 2 shows the most frequently reported obstetric and fetal complications. Placental abruption was reported in three studies, respectively Flemming et al.,^19^ Murthy et al.^26^ and Rasheed et al.^24^ The incidence of placental abruption was reported to be respectively 1%, 6% and 10% in these studies. The incidence of congenital malformations was reported in the study of Hagström et al.^20^ and Salman et al.^18^ to be respectively 2% and 3%. Neither of these studies specified the type of malformations, nor did the rates differ from those reported in non‐cirrhotic pregnancies.

**Table 2:** Fetal and obstetric complications

Study	% caesarean section	% preterm delivery	% postpartum haemorrhage	% pre‐eclampsia	% neonatal mortality	% NICU admission	% small for gestational age	% intrauterine fetal demise	% miscarriage
Salman et al. (2020) ^18^	35.0	25.0		6.0	2.0		7.0	5.0	
Flemming et al. (2020) ^19^	32.7	11.4	7.0				11.0		
Hagström et al. (2018) ^20^	35.9	18.6		3.9	0.9		7.8	0.9	
Jena et al. (2017) ^21^		17.9	17.9	17.9	3.6	21.4	41.7	7.2	57.1
Palatnik & Rinella (2017) ^4^	25.8	45.2	6.5	19.4	6.5	3.2	16.1	3.2	
Danielsson Borssén et al. (2016) ^22^	25.6	23.3							42.4
Puljic et al. (2015) ^23^	69.5	56.8	5.4	13.5	2.7		16.7		
Rasheed et al. (2013) ^24^	81.4	26.4	16.3	12.4	4.7		35.7	0	6.2
Westbrook et al. (2011) ^25^		63.9			0	16.7	22.5	6.5	19.3
Murthy et al. (2009) ^26^	50.4	38.0						0.9	
Britton (1982) 27	13.3				3.6			4.8	10.8

NICU, neonatal intensive care unit.

## 4 DISCUSSION

### 4.1 Main results

Our study demonstrates that women with liver cirrhosis have increased risks of maternal, fetal and obstetric complications during pregnancy and delivery compared with healthy pregnant women. The maternal mortality rate, although lower in recent studies, remained high at 0.89%, whereas in the general obstetric population, maternal mortality is exceedingly rare at 0.010%. Variceal haemorrhage occurred in 4.0% of pregnancies and remained the most common cause of maternal death among women with liver cirrhosis.

### 4.2 Strengths and limitations

To the best of our knowledge, this is the first systematic review and meta‐analysis on this topic. Previous reviews of the literature studied considerably fewer pregnancies, were not available in English or were not systematic reviews.^28^, ^29^, ^30^, ^31^, ^32^ To ensure high quality of evidence and representativeness of the pregnant population with liver cirrhosis, we excluded case reports and case series.

Although in individual studies the quality of selection of cohorts and controls was generally high, selection bias in our study could exist, as included studies used different inclusion criteria. For example, some studies^18^, ^24^ excluded decompensated cirrhosis, leading to an underestimation of reported outcomes.

Before sensitivity analysis, the meta‐analysis showed significant heterogeneity between studies (reasons are given below). The high heterogeneity of the analysis of caesarean section (*I* ^2^ = 68%) cannot be attributed only to differences in known patient characteristics, as is demonstrated by the inability of our sensitivity analyses to lower heterogeneity to an acceptable level. Overall differences in clinical management differences in caesarean section rates between countries are more likely to underlie the heterogeneity. Specifically, there may be distinct regional differences in the management of delivery in liver cirrhosis, some local policies favouring caesarean section to prevent variceal haemorrhage during delivery and other policies favouring vaginal delivery to avoid perioperative risks involved in abdominal surgery.^3^, ^33^

In our meta‐analysis it was not possible to perform subgroup analyses based on diagnosis underlying cirrhosis or severity of cirrhosis, due to the small number of events and missing information in included studies, which could in future studies further allow individualised counselling and management.

### 4.3 Interpretation

The maternal mortality rate in women with liver cirrhosis was 0.89% which is an 80‐fold higher rate compared with pregnant controls without cirrhosis. The most common cause of maternal mortality was variceal haemorrhage during vaginal delivery, although there were large differences in the reported rates of incidence of variceal haemorrhage.^24^ The decrease of variceal haemorrhage in recent studies is probably the result of the inclusion in (inter)national clinical guidelines to screen pregnant women with liver cirrhosis in the second trimester for early detection of oesophageal varices.^34^, ^35^ Oesophageal screening, as currently recommended in 2009 in the American Association for the Study of Liver Diseases guidelines,^36^ may not have been part of routine care during the entire study period of included studies, given the varying rates of endoscopic screening reported in the contributing studies. In addition, treatment options such as endoscopic variceal band ligation have become widely available during the past decades, and were already used in more recent studies included in this systematic review.^4^, ^20^, ^21^ This treatment is considered safe in pregnancy.^37^, ^38^

Our study fulfills the need for expectations when conducting family planning discussions with reproductive women. Cirrhosis is no longer an absolute contraindication to pregnancy, given improved maternal outcomes illustrated in the current study. Related to health status, a more supportive position can be assumed towards women with compensated liver cirrhosis and no history of decompensation, who wish to become pregnant. Previous studies have shown a higher risk of liver‐related complications during pregnancy in women with a history of decompensation^19^, ^24^ or a current Model for End‐stage Liver Disease (MELD) ≥10.^25^ To identify women at risk of variceal haemorrhage, and consequent maternal death, endoscopic screening for oesophageal varices in the 12 months preceding conception could be of value. Medium or large oesophageal varices should be treated, preferably before pregnancy, by variceal band ligation.^35^

The risk of mortality in both pregnant and non‐pregnant patients with cirrhosis depends on aetiology, severity and presence of complications, as well as the presence of comorbid conditions.^39^ The reported overall 30‐day mortality rate after an episode of variceal bleeding is 15–20%,^8^ which is similar with the mortality of 12% after a variceal bleed in pregnant women in our study. The 1‐year mortality of non‐pregnant patients with compensated liver cirrhosis is 1.0% and that of patients with compensated liver cirrhosis as well as oesophageal varices 3.4%. These data apply to all cirrhosis patients, but 67% of patients with cirrhosis are males older than 50 years.^40^ In comparison, the maternal mortality rates (<1%) among pregnant cirrhosis patients may be slightly lower, likely owing to the comparatively young age of pregnant women. Due to our study design, we were not able to investigate whether pregnancy is associated with more rapid progression of cirrhosis than can be expected according to age.

The meta‐analysis showed significant heterogeneity, mostly attributed to two studies (Flemming et al.^19^ and Rasheed et al.^24^) as demonstrated by our sensitivity analysis. The study of Rasheed et al. differs in various aspects from the other included studies. It is, in contrast to most other included studies, a prospective cohort study in a middle‐income country, possibly reflecting a lower level of care. This study included all pregnancies including miscarriages, whereas the other studies only described deliveries. Moreover, the aetiology of liver cirrhosis, in this study solely viral hepatitis, differs from those in the other studies, which included various causes. The study of Flemming et al. did not have major differences on study design, study population or aetiology of liver cirrhosis. However, their case definition differed because their analysis evaluated only primiparous women.

## 5 CONCLUSION

This is the first systematic review and meta‐analysis of pregnancy in women with liver cirrhosis. Liver cirrhosis complicates pregnancies for both mother and child.

### 5.1 Implications for research

While our systematic review was able to quantify the increased risks of liver cirrhosis in pregnancy, we were insufficiently powered to demonstrate whether emerging management options have lowered some or all of these risks. Our study was not able to provide evidence on the patient selection most likely to benefit from such management options. In particular, more data regarding course and treatment of liver cirrhosis during pregnancy and treatment of specific complications (e.g. variceal haemorrhage, ascites, jaundice, hepatic encephalopathy) are needed to improve the knowledge and management of women with these health issues and to identify the women more at risk. We suggest large prospective international studies could provide evidence for many of these knowledge gaps and could investigate which diagnostic and treatment entities could contribute to improved perinatal outcomes over time.

### 5.2 Implications for practice

This systematic review provides evidence‐based expectations for clinicians and pregnant women with cirrhosis and will improve pregnancy management for pregnant women with liver cirrhosis.

## AUTHOR CONTRIBUTION

The study was conceived by RCP and RBT. FSE performed the literature search. LLS and RBT carried out selection of eligible studies. LLS and IS extracted data and carried out the data analysis. RCP supervised the data analysis. Differences of opinion were resolved by consensus with RCP. All authors were involved in interpretation of data and revised the article critically.

## CONFLICT OF INTERESTS

None declared. Completed disclosure of interest forms are available to view online as supporting information.

## ETHICAL APPROVAL

None.

## Supporting information


**Appendix** S1

Figure S1

Figure S2

Table S1

Table S2

Data S1

Data S2

Data S3

Data S4

Data S5

## Data Availability

Data sharing not applicable to this article as no datasets were generated or analysed during the current study
