# Supplementary material for: Pregnancy in women with liver cirrhosis is associated with increased risk for complications: A systematic review and meta‐analysis of the literature
Source: BJOG. 2022 Mar 31;129(10):1644–52. doi: 10.1111/1471-0528.17156 (PMC9546282; doi:10.1111/1471-0528.17156)
Supplement: Supplementary file 1 — Appendix S1 [file BJO-129-1644-s006.docx]

**Appendix S1** Search strategy

**Database 1: PUBMED**

| **#** | **Searches** |
| --- | --- |
| 1 | ("Liver Cirrhosis"[Mesh] OR "Hypertension, Portal"[Mesh] OR "Hepatic Insufficiency"[Mesh] OR "Hepatitis, Autoimmune"[Mesh] OR "Liver Diseases, Alcoholic"[Mesh] OR "Hepatitis B, Chronic"[Mesh] OR "Hepatitis C, Chronic"[Mesh] OR liver cirrhosis[tiab] OR portal hypertension[tiab] OR autoimmune hepatitis[tiab] OR biliary liver cirrhosis[tiab] OR primary biliary cirrhosis[tiab] OR primary biliary cholangitis[tiab] OR ductal plate malformation[tiab] OR congenital liver fibrosis[tiab] OR chronic hepatitis[tiab] OR alcoholic liver disease[tiab] OR oesophageal varices[tiab] OR esophageal varices[tiab] OR hepatic insufficiency[tiab] OR liver failure [tiab] OR chronic liver disease[tiab]) |
| 2 | ("Pregnant Women"[Mesh] OR "Pregnancy"[Mesh] OR pregnan*[tiab]) |
| 3 | ("Pregnancy Complications"[Mesh] OR complication*[tiab] OR (pregnan*[tiab] AND outcome*[tiab])) AND |
| 4 | #1 and #2 and #3 |

**Database 2: EMBASE (Ovid)**

| **#** | **Searches** |
| --- | --- |
| 1 | exp *liver cirrhosis/ or *portal hypertension/ or *liver failure/ or autoimmune hepatitis/ or exp alcohol liver disease/ or *chronic hepatitis B/ or *chronic hepatitis C/ or (liver cirrhosis or portal hypertension or autoimmune hepatitis or biliary liver cirrhosis or primary biliary cirrhosis or primary biliary cholangitis or ductal plate malformation or congenital liver fibrosis or chronic hepatitis or alcoholic liver disease or oesophageal varices or esophageal varices or hepatic insufficiency or liver failure or chronic liver disease).ti,ab,kw. |
| 2 | pregnant woman/ or exp pregnancy/ or pregnan*.ti,ab,kw. |
| 3 | exp pregnancy complication/ or exp complication/ or complication.fs. or complication*.ti,ab,kw. or (pregnan* and outcome*).ti,ab,kw. |
| 4 | #1 and #2 and #3 |
