# Supplementary figures and images for: Pregnancy in women with liver cirrhosis is associated with increased risk for complications: A systematic review and meta‐analysis of the literature
Source: BJOG. 2022 Mar 31;129(10):1644–52. doi: 10.1111/1471-0528.17156 (PMC9546282; doi:10.1111/1471-0528.17156)

# Incidence of maternal mortality

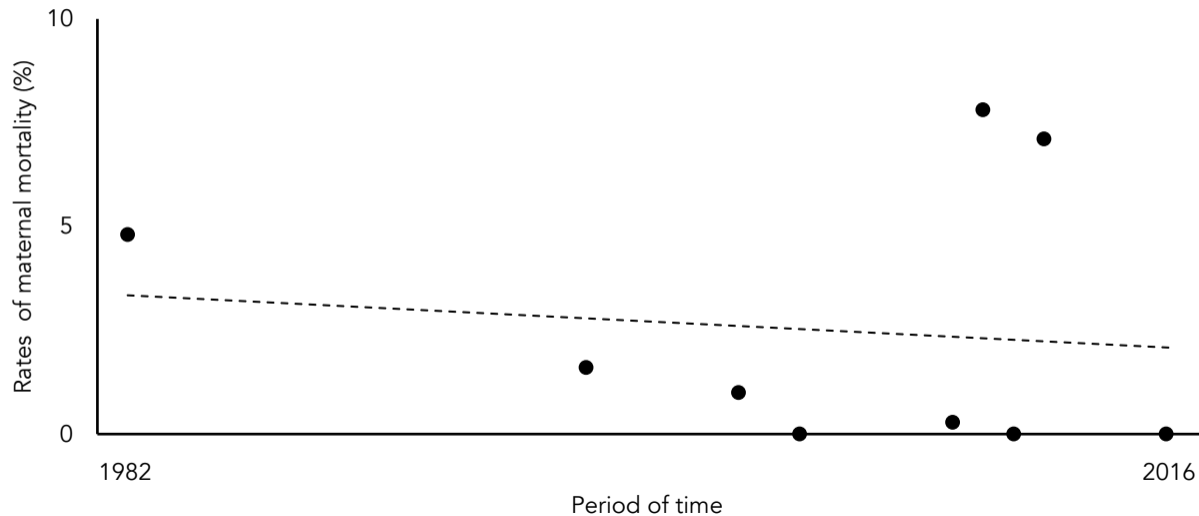

Supplement: Supplementary file 2 — Figure S1 [file BJO-129-1644-s001.pdf]

# Incidence of variceal hemorrhage

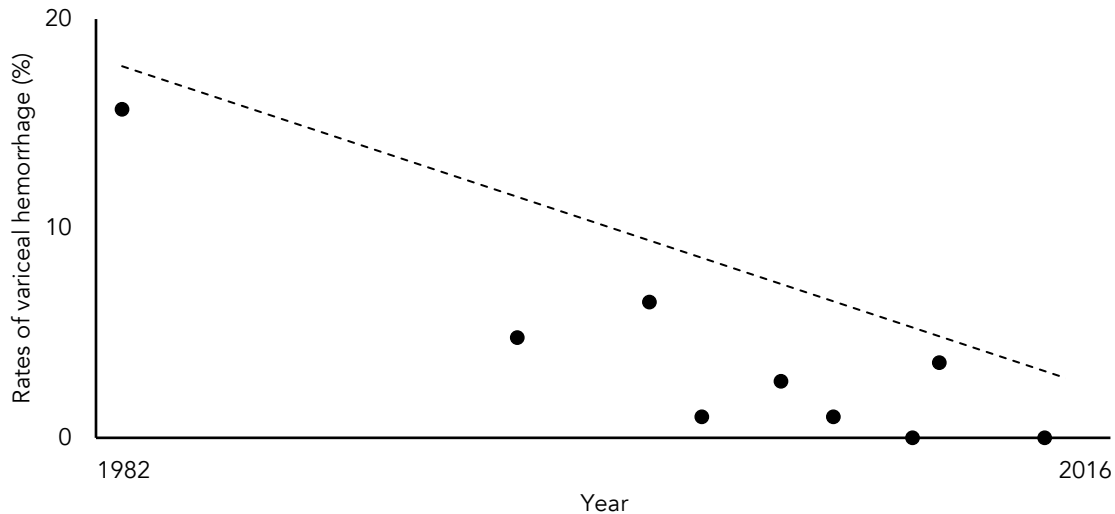

Supplement: Supplementary file 3 — Figure S2 [file BJO-129-1644-s007.pdf]
