# Supplementary material for: Pregnancy in women with liver cirrhosis is associated with increased risk for complications: A systematic review and meta‐analysis of the literature
Source: BJOG. 2022 Mar 31;129(10):1644–52. doi: 10.1111/1471-0528.17156 (PMC9546282; doi:10.1111/1471-0528.17156)
Supplement: Supplementary file 4 — Table S1 [file BJO-129-1644-s005.pdf]

Table S1. Quality assessement

First author, year	Selection				Comparability	Outcome			Total	Quality
	**A**	**B**	**C**	**D**	**E**	**F**	**G**	**H***		
Salman (2020)	★	★	★	★	★	★	★	★	8	Good quality
Flemming (2020)	★	★	★	★	★★	★	★		8	Good quality
Hagström (2018)	★	★	★	★	★★	★	★		8	Good quality
Jena (2017)	★		★	★		★	★		5	Fair quality
Palatnik (2016)	★	★	★	★	★★		★		8	Good quality
Borssen (2016)	★			★		★	★		3	Poor quality
Puljic (2015)	★	★	★	★	★★	★	★		8	Good quality
Rasheed (2013)	★	★	★	★	★★	★	★	★	9	Good quality
Westbook (2011)	★		★	★		★	★		5	Fair quality
Murthy (2009)	★	★	★	★	★	★	★		7	Fair quality
Britton (1984)	★		★	★		★	★		5	Fair quality

A: Representativeness of the exposed cohort. B: Selection of the non-exposed cohort. C: Ascertainment of exposure. D: Outcome of interest not present at start study. E: Comparability of cohorts on the basis of the design or analysis: study controls for non-cirrhotic pregnant controls (one point) and for age, body mass index, parity or year of delivery (two points). F: Assessment of outcome. G: Follow-up long enough for outcomes to occur. H: Adequacy of follow up of cohorts: lost to follow up <20%. *Included studies were all retrospective cohorts, except [18,24].
