# Supplementary material for: Pregnancy in women with liver cirrhosis is associated with increased risk for complications: A systematic review and meta‐analysis of the literature
Source: BJOG. 2022 Mar 31;129(10):1644–52. doi: 10.1111/1471-0528.17156 (PMC9546282; doi:10.1111/1471-0528.17156)
Supplement: Supplementary file 5 — Table S2 [file BJO-129-1644-s009.docx]

**Table S2.** Causes of maternal death.

| **Causes** | **Total (n=25)** |
| --- | --- |
| Variceal hemorrhage | n=14 |
| during vaginal delivery | n=9 |
| during pregnancy | n=2 |
| during cesarean section | n=1 |
| postpartum | n=1 |
| Unknown | n=6 |
| Sepsis | n=2 |
| Preeclampsia | n=1 |
| Hepatic decompensation | n=2 |
| Flare of autoimmune hepatitis | n=1 |
